# Author reply

**DOI:** 10.1192/bjb.2019.28

**Published:** 2019-06

**Authors:** John L. Taylor

**Affiliations:** Professor of Clinical Psychology, Northumbria University, UK. email: john.taylor@ntw.nhs.uk

Jean O'Hara is correct in saying that I am a responsible clinician working with people with intellectual disabilities with offending histories and complex needs – in both in-patient and community settings. As such, my experience of the impact of the Transforming Care national plan is concrete and real rather than ‘perceptual’.

It is nonetheless encouraging to learn that NHS England recognises that some people with intellectual disabilities and/or autism need high-quality in-patient care and treatment at times. The question is, post Transforming Care, where are they going to receive it given the wholesale closure of specialist NHS services – including some rated by the Care Quality Commission as outstanding? The options would appear to be either the profit-focused private sector, or acute mental health in-patient units where the specialist care and treatment required is not available.

I note that Dr O'Hara doesn't refer to the data that indicate clearly that the Transforming Care national plan has failed to significantly reduce the number of people with intellectual disabilities and/or autism in in-patient facilities. Rather, she introduces a new metric of the ‘need for admission’. This is not defined but seems to refer to admission rates. It is suggested that the national plan has been successful in reducing the variation in the ‘need for admission’ across the country. Given that a reduction in the geographical variation of admission rates could be achieved by closing beds in some areas whilst maintaining (or increasing) bed numbers in others, with no overall reduction in the number of beds across the country, one might wonder whether this is another case of smoke and mirrors?

Three-and-a-half years on, the Transforming Care programme has failed to invest the tens of millions of pounds in community services in England promised in the national plan. Can we then be comforted by the news that the NHS Long Term Plan ‘is explicit about its focus on increasing investment in intensive, crises and community support’, or is this just another example of ‘jam tomorrow and jam yesterday, but never jam today’^1^ for people with intellectual disabilities?
